# Maternal and perinatal outcomes of women with vaginal birth after cesarean section compared to repeat cesarean birth in select South Asian and Latin American settings of the global network for women’s and children’s health research

**DOI:** 10.1186/s40748-023-00169-x

**Published:** 2023-11-01

**Authors:** Lester Figueroa, Margo Harrison, Manolo Mazariegos, Shivaprasad Goudar, Avinash Kavi, Richard Derman, Archana Patel, Prabir Das, Patricia L. Hibberd, Sarah Saleem, Farnaz Naqvi, Robert L. Goldenberg, Rashidul Haque, Sk Masum Billah, William A. Petri, Elizabeth M. McClure, Sylvia Tan, Nancy F. Krebs

**Affiliations:** 1 Instituto de Nutrición de Centroamérica y Panamá –INCAP, Calzada Roosevelt 6-25 zona 11, C.A Guatemala City, Guatemala; 2 University of Colorado School of Medicine, Denver, CO USA; 3 KLE University’s Jawaharlal Nehru Medical College, Belgaum, India; 4 Thomas Jefferson University, Philadelphia, PA USA; 5 Lata Medical Research Foundation, Nagpur, India; 6 Datta Meghe Institute of Medical Sciences, Wardha, India; 7 Boston University, Boston, MA USA; 8 Department of Community Health Sciences, Aga Khan University, Karachi, Pakistan; 9 Department of Obstetrics and Gynecology, Columbia University School of Medicine, New York, NY USA; 10 International Centre for Diarrhoeal Disease Research, Bangladesh (icddr, b), Dhaka, Bangladesh; 11 Sydney School of Public Health, The University of Sydney, Sydney, NSW Australia; 12 University of Virginia, Charlottesville, VG USA; 13 RTI International, Durham, NC USA

**Keywords:** Mode of birth, Cesarean birth, Repeat cesarean birth, Low- and middle-income countries, Facility-based delivery, Maternal outcomes, Neonatal outcomes, Vaginal birth after cesarean, Breastfeeding initiation

## Abstract

**Objective:**

Our objective was to analyze a prospective population-based registry including five sites in four low- and middle-income countries to observe characteristics associated with vaginal birth after cesarean versus repeat cesarean birth, as well as maternal and newborn outcomes associated with the mode of birth among women with a history of prior cesarean.

**Hypothesis:**

Maternal and perinatal outcomes among vaginal birth after cesarean section will be similar to those among recurrent cesarean birth.

**Methods:**

A prospective population-based study, including home and facility births among women enrolled from 2017 to 2020, was performed in communities in Guatemala, India (Belagavi and Nagpur), Pakistan, and Bangladesh. Women were enrolled during pregnancy, and delivery outcome data were collected within 42 days after birth.

**Results:**

We analyzed 8267 women with a history of prior cesarean birth; 1389 (16.8%) experienced vaginal birth after cesarean, and 6878 (83.2%) delivered by a repeat cesarean birth. Having a repeat cesarean birth was negatively associated with a need for curettage (ARR 0.12 [0.06, 0.25]) but was positively associated with having a blood transfusion (ARR 3.74 [2.48, 5.63]). Having a repeat cesarean birth was negatively associated with stillbirth (ARR 0.24 [0.15, 0.49]) and, breast-feeding within an hour of birth (ARR 0.39 [0.30, 0.50]), but positively associated with use of antibiotics (ARR 1.51 [1.20, 1.91]).

**Conclusions:**

In select South Asian and Latin American low- and middle-income sites, women with a history of prior cesarean birth were 5 times more likely to deliver by cesarean birth in the hospital setting. Those who delivered vaginally had less complicated pregnancy and labor courses compared to those who delivered by repeat cesarean birth, but they had an increased risk of stillbirth. More large scale studies are needed in Low Income Country settings to give stronger recommendations.

**Trial registration:**

NCT01073475, Registered February 21, 2010, https://clinicaltrials.gov/ct2/show/record/NCT01073475.

## Synopsis

Repeat cesarean birth in select Latin American and South Asian sites was the more common mode of birth and was associated with reduced stillbirth but also with reduced postpartum breastfeeding, and increased infant antibiotic and oxygen use compared to vaginal birth after cesarean.

## Introduction

Cesarean birth rates are rising globally [1–3]. Women who have had a previous cesarean birth may be able to choose a repeat cesarean birth (RCB) or a vaginal birth after cesarean (VBAC) if both options are available [4–6]. Both modes of birth have risks and benefits, but appropriately selected women can often successfully achieve a vaginal birth after cesarean without an undue burden of adverse maternal and neonatal outcomes if they do so in a setting capable of managing complications [6–8]. In high-income countries, vaginal birth after cesarean (VBAC) rates have slowly increase with a concurrent reduction in RCB rates [1, 8–10]. The balance in these settings often favors RCB for pregnancy outcomes such as reduced stillbirth, while VBAC favors maternal outcomes, such as reduced hemorrhage and postpartum thrombotic events [7, 11, 12]. However, it is vital to recognize that the decision regarding the mode of birth, particularly repeated cesarean sections, goes beyond medical considerations and enters the complex medico-legal landscape. In the context of repeated cesarean sections, where a woman chooses this procedure without a medical indication, several factors come into play, including concerns about medical malpractice lawsuits and the medicalization of childbirth [13].

In low- and middle-income countries, mode of birth among women with a history of prior cesarean varies by region [1, 8]. In sub-Saharan Africa, there is generally a lack of access to cesarean birth [14–16], which means that even if a woman underwent a cesarean for a prior birth, she might not have access to a repeat procedure in subsequent pregnancies and therefore, the decision to pursue the trial of labor after a cesarean birth may be her only option. In the African setting, lack of access to high-quality emergency obstetric care may result in adverse outcomes for mothers and babies [14–17]. In other low- and middle-income countries where there is greater access to emergency obstetric care, most births following a cesarean occur by RCB, with a minority of VBAC [18–20].

This topic is of interest because as cesarean birth rates rise, accompanied by the adverse outcomes associated with major abdominal surgery, safely attempting vaginal birth after cesarean with trained providers in an appropriate setting may be one method of curbing rising global cesarean birth rates [20]. Our objective was to analyze a prospective population-based pregnancy outcome registry from five sites in four low- and middle-income countries to observe characteristics associated with the mode of birth among women with a history of prior cesarean, as well as to examine the pregnancy outcomes of those births. We hypothesized that maternal and perinatal outcomes among women who undergo vaginal births after cesarean section (VBAC) will be comparable to those among women who opt for recurrent cesarean births.

The novel aspect of our research is rooted in the inclusion of data from a prospective population-based maternal registry in low- and middle-income countries with limited data, which offers a unique perspective on the topic. While our study acknowledges the existing body of research on this topic, it seeks to provide a fresh and valuable contribution to the field.

## Materials and methods

This secondary analysis was conducted using data from a prospective study conducted in communities at eight sites in seven low-income countries on births 2017 through 2020 in the sites of the Global Network for Women’s and Children’s Health Research (GN), which is a NICHD-funded network studying pregnancy outcomes in low- and middle-income settings [19]. Data in this report represent those from the Guatemala, India (Belagavi and Nagpur), Bangladesh, and Pakistan sites. Data were also collected at three sites in sub-Saharan Africa (Kenya, Zambia, Democratic Republic of Congo), but because the cesarean birth rates in these sites are very low, the data from these settings were not included.

The GN’s prospective registry, the Maternal and Newborn Health Registry (MNHR), includes outcomes from sites that generally include between 6 and 12 communities. About 300 to 500 births take place annually in each community, which is usually served by a primary healthcare center. The intent of designing the MNHR was to enroll pregnant women and to obtain data on pregnancy outcomes for all deliveries of registered women, regardless of delivery location.

**Fig. 1:**
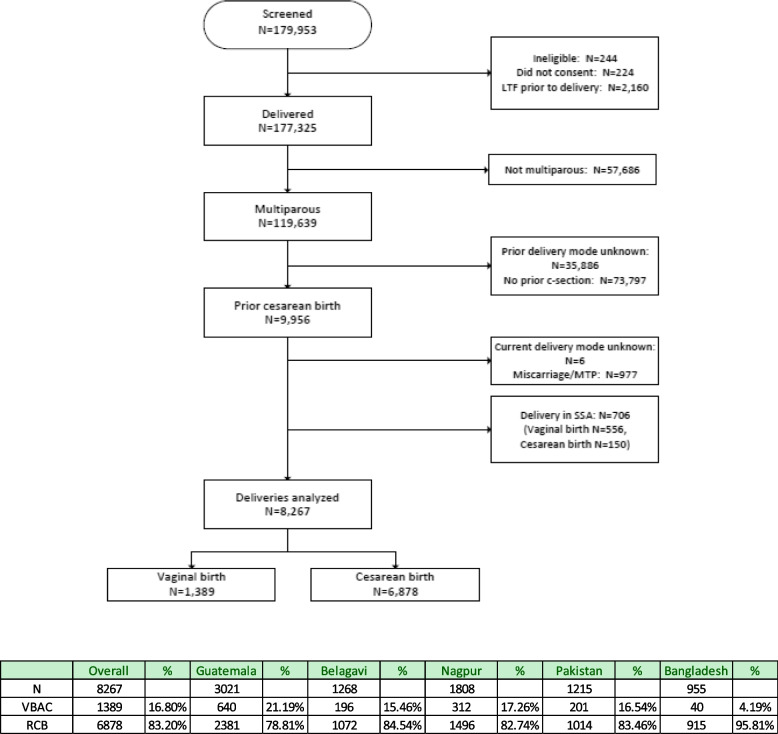
Consort Diagram Association of Maternal and Obstetric characteristics with mode of birth (VBAC versus repeat cesarean birth), among births at Selected Sites (Guatemala, Belagavi, Nagpur, Pakistan and Bangladesh) at the Global Network for Women’s and Children’s Health Research, Maternal and Newborn Health Registry, 2017-2020

MNHR staff primarily comprise community workers and nurses who are assigned specific geographical areas. Their responsibilities involve conducting three crucial visits: The first visit, known as the enrollment visit, is ideally scheduled during the first trimester of pregnancy. During this visit, they meticulously gather essential pregnancy-related data. The second visit is conducted within the first 48 h postpartum, where they collect comprehensive birth-related data, ensuring timely and accurate documentation and finally, the third visit takes place 42 days postpartum, during which they focus on gathering morbidity and mortality data [21].

The population studied included women screened for the MNHR who were eligible, consented, and delivered between 2017 and 2020. Data were excluded for women who were enrolled but lost to follow-up prior to delivery, maternal deaths prior to labor and delivery, miscarriages, medically terminated pregnancies, pregnancies complicated by breech or other malpresentations, and those with missing data for delivery.

Data were collected and transmitted through secure methods to a central data-coordinating center (RTI International). Counts and percentages of modes of birth were obtained using standard contingency table techniques. Bivariate comparisons of women achieving vaginal birth after cesarean compared to RCB were assessed. A multivariate model of VBAC compared to RCB was assessed with a priori selected covariates of delivery location, maternal hypertensive disease, dysfunctional labor, maternal schooling, and maternal age. Subsequently, individual models of maternal and perinatal outcomes were fit to determine which outcomes were more and less common among women pursuing RCB as compared to vaginal birth after cesarean. Each of these models was adjusted for the characteristics that were predictive of RCB compared with vaginal birth after cesarean at *p* < 0.1. All statistical comparisons were performed using robust Poisson regressions, adjusting for the correlation within cluster. Statistical analyses were performed using SAS software version 9.4 (SAS Institute, Cary, NC, USA).

The appropriate institutional review boards/ethics research committees of the participating institutions approved the MNHR study. Individual informed consent for study participation is requested and obtained from each study participant. A Data Monitoring Committee, appointed by the NICHD, oversees, and reviews the study semi-annually.

## Results

Figure 1 is a consort diagram of our study population. Among women with a history of prior cesarean birth across the GN sites (*n* = 8267), 1389 (16.8%) experienced vaginal birth after cesarean, and 6878 (83.2%) delivered by a RCB. The VBAC rate ranged from 4.2% in Bangladesh, 15.5% in Belagavi, 17.3% in Nagpur, 16.5% in Pakistan, up to 21.2% in Guatemala.

Compared to those who underwent RCB, women in these selected GN sites who achieved VBAC have differed in terms of education (7.8% vs. 13.8% achieved higher education), parity (29.1% vs. 9.4% had parity > 2), body mass index (BMI) (35.7% vs. 40.3% had BMI ≥ 25), median inter-delivery interval (32 vs. 37 months), number of antenatal care visits (59.1% vs. 72.7% had ≥ 4 visits), experiencing obstructed labor or failure to progress (2.9% vs. 8.7%), experiencing hypertensive disorders (2.2% vs. 5.7%), undergoing induction of labor (3.6% vs. 0.5%), choice of delivery location (51.7% vs. 83.2% in the hospital), and preterm birth rates (20.0% vs. 16.6%) (Table 1).

**Table 1:** Association of maternal and obstetric characteristics with mode of birth (VBAC versus RCB), among births at selected sites (Guatemala, Belagavi, Nagpur, Pakistan and Bangladesh) at the global network for Women’s and Children’s health research, maternal and newborn health registry, 2017–2020

Characteristics	Women with a history of cesarean birth	VBAC	RCB	*P*-value*
**Mothers, N**	**8,267**	**1,389**	**6,878**	
Maternal age, n	8266.0	1389.0	6877.0	0.0811
median (P25, P75)	26.0 (24.0, 30.0)	27.0 (24.0, 30.0)	26.0 (23.0, 30.0)	.
Maternal education, n (%)	8,266	1,389	6,877	< .0001
No formal schooling	1,114 (13.5)	269 (19.4)	845 (12.3)	
Primary or secondary	6,092 (73.7)	1,012 (72.9)	5,080 (73.9)	
University +	1,060 (12.8)	108 (7.8)	952 (13.8)	
Parity	8,267	1,389	6,878	< .0001
1	5,229 (63.3)	634 (45.6)	4,595 (66.8)	
2	1,990 (24.1)	351 (25.3)	1,639 (23.8)	
> 2	1,048 (12.7)	404 (29.1)	644 (9.4)	
BMI kg/m^2^	8,257	1,388	6,869	<.0001
< 18.5	1,093 (13.2)	201 (14.5)	892 (13.0)	
18.5–24.9	3,899 (47.2)	692 (49.9)	3,207 (46.7)	
≥ 25	3,265 (39.5)	495 (35.7)	2,770 (40.3)	
Inter-delivery interval, n	8258.0	1388.0	6870.0	<.0001
median (P25, P75)	36.0 (25.0, 53.0)	32.0 (23.0, 47.0)	37.0 (25.0, 55.0)	
At least one antenatal care visit, n/N (%)	7,879/8,267 (95.3)	1,314/1,389 (94.6)	6,565/6,878 (95.4)	<.0001
Antenatal care visits, n/N (%)	8,257	1,388	6,869	<.0001
0–3	2,441 (29.6)	568 (40.9)	1,873 (27.3)	
≥ 4	5,816 (70.4)	820 (59.1)	4,996 (72.7)	
Obstructed labor/failure to progress, n/N (%)	636/8,266 (7.7)	40/1,389 (2.9)	596/6,877 (8.7)	0.0061
Severe antepartum hemorrhage, n/N (%)	61/8,267 (0.7)	13/1,389 (0.9)	48/6,878 (0.7)	0.1308
Hypertensive disorders, n/N (%)	422/8,267 (5.1)	30/1,389 (2.2)	392/6,878 (5.7)	0.0005
Induction of labor, n/N (%)	80/7,967 (1.0)	48/1,334 (3.6)	32/6,633 (0.5)	< .0001
Delivery attendant**, n (%)	8,267	1,389	6,878	**
Physician	7,515 (90.9)	637 (45.9)	6,878 (100.0)	
Nurse/nurse midwife/LHW/HW	227 (2.7)	227 (16.3)	0 (0.0)	
Traditional birth attendant	481 (5.8)	481 (34.6)	0 (0.0)	
Family/self/other	44 (0.5)	44 (3.2)	0 (0.0)	
Delivery Location, n (%)	8,267	1,389	6,878	< .0001
Hospital	6,442 (77.9)	718 (51.7)	5,724 (83.2)	
Clinic/Health center	1,123 (13.6)	164 (11.8)	959 (13.9)	
Private Center	702 (8.5)	507 (36.5)	195 (2.8)	
**Infants, N**	**8,335**	**1,398**	**6,937**	
Female sex, n (%)	4,110/8,302 (49.5)	696/1,367 (50.9)	3,414/6,935 (49.2)	0.2942
Preterm birth, n (%)	1,432/8,325 (17.2)	277/1,388 (20.0)	1,155/6,937 (16.6)	0.0054
Measured birth weight, n	8258	1337	6921	0.0979
median (P25, P75)	2800.0 (2500.0, 3010.0)	2780.0 (2500.0, 3030.0)	2800.0 (2500.0, 3010.0)	

* With the exception of measured birth weight, all analyses are performed using robust Poisson regression, and p-values are adjusted for the correlation within study cluster. The comparisons of measured birth weight are performed using a Wilcoxon nonparametric test

** Statistical analysis not performed due to the lack of variation in the RCB group

In multivariate modeling using a priori selected characteristics, those that were associated with the likelihood of delivering by VBAC as compared to RCB were hospital delivery versus other location (ARR 0.2 [0.2,0.3]); having a hypertensive disorder (ARR 0.6 [0.4,0.8]); experiencing obstructed labor or failure to progress (ARR 0.4 [ 0.2,0.9]); and having schooling versus no formal education or being illiterate (ARR 0.7 [0.6,0.8]) (Table 2).

**Table 2:**
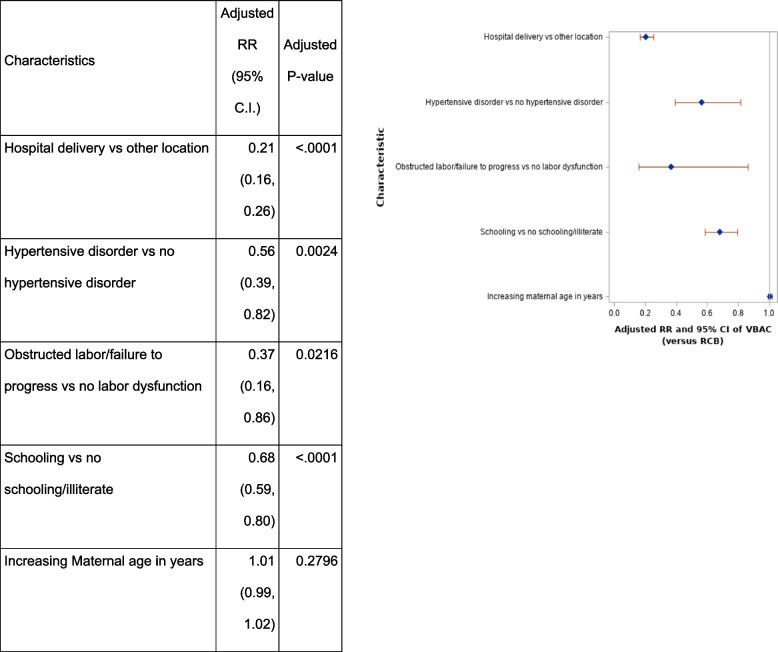
Adjusted association of maternal and obstetric characteristics with mode of birth (VBAC versus RCB ), among births at selected sites (Guatemala, Belagavi, Nagpur, Pakistan and Bangladesh) at the global network for Women’s and Children’s health research, maternal and newborn health registry, 2017-2020

The analysis is performed using robust Poisson regression; Relative risks (RR) and p-values are jointly adjusted for the presence of all other factors in the model and for the correlation within study cluster. The reference is RCB

Table 3, presents the association between maternal and neonatal outcomes and mode of birth among women with a history of previous cesarean birth adjusted for age, education, BMI, hypertensive disease, birth interval, parity, number of antenatal care visits, obstructed labor, delivery location, and preterm birth. Models for infant outcomes were additionally adjusted for birthweight. Compared to women who achieved VBAC, those who underwent RCB had a higher risk of blood transfusion (8.4% versus 1.5%, ARR 3.7 [2.5,5.6]) and a lower risk of dilation and curettage or a suction procedure (0.2% versus 2.4%, ARR 0.1 [0.1,0.3]). In terms of perinatal outcomes, women undergoing RCB had a lower risk of stillbirth (11% versus 72%, ARR 0.3 [0.2, 0.5]) and a lower likelihood of breastfeeding within one hour (26% versus 72%, ARR 0.4 [0.3, 0.5]) as compared to women giving birth vaginally. However, RCB was associated with greater likelihood of requiring infant antibiotics (9.3% versus 5.3%, ARR 1.5 [1.2, 1.9]) and supplemental oxygen (8% versus 5%, ARR 1.3 [1.02, 1.67]).

**Table 3:** Risk of outcomes by mode of delivery (RCB vs. VBAC) from selected Sites (Guatemala, Belagavi, Nagpur, Pakistan and Bangladesh) of global network for Women’s and Children’s health research, maternal and newborn health registry

Characteristics	Women with a history of cesarean birth	VBAC	RCB	*P*-value*	Adjusted** *P*-value	Adjusted** RR (95% C.I.)
**Mothers, N**	**8,267**	**1,389**	**6,878**			
Severe postpartum hemorrhage, n (%)	80/8,259 (1.0)	19/1,389 (1.4)	61/6,870 (0.9)	0.0577	0.0683	0.55 (0.29, 1.05)
Uterotonics, n (%)	6,685/8,237 (81.2)	845/1,388 (60.9)	5,840/6,849 (85.3)	0.0003	0.2384	1.07 (0.96, 1.19)
Blood transfusion, n (%)	602/8,266 (7.3)	21/1,389 (1.5)	581/6,877 (8.4)	< .0001	< .0001	3.74 (2.48, 5.63)
D&C or suction, n (%)	47/8,265 (0.6)	33/1,389 (2.4)	14/6,876 (0.2)	< .0001	< .0001	0.12 (0.06, 0.25)
Magnesium Sulfate, n (%)	205/8,232 (2.5)	7/1,388 (0.5)	198/6,844 (2.9)	0.0304	0.0748	1.93 (0.94, 3.99)
Hysterectomy, n (%)	28/8,265 (0.3)	1/1,389 (0.1)	27/6,876 (0.4)	0.1000	0.0757	10.54 (0.78, 141.70)
Severe perinatal infection, n (%)	112/8,266 (1.4)	18/1,389 (1.3)	94/6,877 (1.4)	0.7226	0.9015	1.03 (0.64, 1.66)
Severe postnatal infection/sepsis, n (%)	37/8,258 (0.4)	3/1,386 (0.2)	34/6,872 (0.5)	0.2291	0.1058	2.79 (0.80, 9.70)
Seizures/coma, n (%)	15/8,258 (0.2)	0/1,386 (0.0)	15/6,872 (0.2)	***	***	***
Unplanned hospitalization, n (%)	776/8,265 (9.4)	97/1,389 (7.0)	679/6,876 (9.9)	0.0272	0.9359	0.99 (0.79, 1.24)
Maternal death < 42 days, rate per 100,000 births	9 (109)	4 (288)	5 (73)	0.0301	***	***
**Infants, N**	**8,335**	**1,398**	**6,937**			
Stillbirths, rate per 1,000 stillbirths plus live births	176 (21.1)	100 (72.0)	76 (11.0)	< .0001	< .0001	0.27 (0.15, 0.49)
Bag and mask resuscitation	395/8,298 (4.8)	60/1,372 (4.4)	335/6,926 (4.8)	0.6114	0.8938	1.02 (0.72, 1.45)
Baby breastfed within an hour	2,699/8,148 (33.1)	926/1,289 (71.8)	1,773/6,859 (25.8)	< .0001	< .0001	0.39 (0.30, 0.50)
Antibiotics	716/8,308 (8.6)	73/1,377 (5.3)	643/6,931 (9.3)	0.0003	0.0005	1.51 (1.20, 1.91)
CPAP	44/8,301 (0.5)	5/1,375 (0.4)	39/6,926 (0.6)	0.3936	***	***
Oxygen	647/8,306 (7.8)	75/1,378 (5.4)	572/6,928 (8.3)	0.0027	0.0319	1.31 (1.02, 1.67)
Neonatal mortality < 28 days, rate per 1,000 live births	185 (22.7)	54 (42.0)	131 (19.1)	< .0001	0.4734	0.86 (0.57, 1.30)

* All analyses are performed using robust Poisson regression; All p-values are adjusted for the correlation within study cluster.** The relative risk is expressed as the risk of each outcome for RCB against the reference of VBAC. The relative risk is additionally adjusted for the factors in Table 1b that were significant at *P* < 0.1: maternal age, education, BMI, hypertension, interdelivery interval, parity, number of ANC visits, obstructed labor, delivery location, preterm birth; models of infant outcomes are additionally adjusted for measured birth weight.*** Analyses could not be performed due to zero or small cell sizes

## Discussion

In our analysis of women participating in South Asian and Latin American sites of the GN MNHR maternal and neonatal health registry who had a history of prior cesarean birth, the majority underwent RCB with rates ranging from 78.8% in Guatemala to 95.8% in Bangladesh. Maternal hypertensive disease and obstructed labor were predictive of RCB in adjusted modeling. Adjusted modeling also revealed RCB was associated with a higher risk of blood transfusion but a lower likelihood of needing a dilation and curettage.

RCB was also associated with lower adjusted rates of stillbirths compared to VBAC. More women with VBAC gave birth in the home setting (36.5%) where no fetal monitoring was available, compared to women with RCB (2.8%).

Lower rates of breastfeeding initiation within the first hour after delivery were observed for those with RCB, an outcome with implications for both maternal and infant health.

Our findings are consistent with prior studies [1, 7–19], which have also shown that women undergoing cesarean birth had a greater likelihood of postpartum maternal blood transfusion but were less likely to need dilation and curettage. Overall, the prevalence of blood transfusion and dilation and curettage in developing countries are low as described in other studies [1, 13, 15], but this is partially explained by the inequity of access to maternal health.

While these findings are consistent with other literature, it is notable that the rate of VBAC in Bangladesh is less than 5%. This is even lower than the rate in high-income settings and is certainly an outlier even among our study sites with lower cesarean birth rates. Recent data show that our findings are consistent with the rise of cesarean rates [5, 22–24], and VBAC could be a safe intervention to lower this rate [6, 25]. However, it’s essential to note that making definitive statements regarding the safety of VBAC would require a more comprehensive dataset, encompassing complications such as uterine rupture in VBAC [1] and complications like Isthmocele in RCB [26]. Another finding is that babies born via VBAC were less likely to receive antibiotics and supplemental oxygen, and were more likely to be breastfed within one hour of birth. This is important since delayed breastfeeding initiation may harm neonatal health and survival, including infection associated with neonatal mortality [24].

It is known that babies born by RCB have a higher risk of needing supplemental oxygen due to transient tachypnea of the newborn. Also, significantly greater usage of antibiotics has previously been reported for infants born via cesarean delivery [7, 11, 25], but a limitation of our data is that the indication for the administration of oxygen was not documented.

The impact of cesarean delivery on delayed initiation of breastfeeding has been previously reported for low- and middle-income countries [26–30]. The data presented in the current analysis point to an opportunity to counter this by VBAC or by anticipation of the effects of RCB on breastfeeding and to encourage perinatal interventions to support breastfeeding initiation.

## Conclusions

The high prevalence of RCBs in some countries, such as Bangladesh, highlights the need for research on ways to safely and successfully promote vaginal birth after cesarean (VBAC) in low-resource settings. The increasing rates of cesarean birth can strain the healthcare system and have negative impacts on the health and well-being of women and their offspring. This is especially concerning in settings where there may be limited resources and higher rates of morbidity and mortality. The findings of this analysis align with existing literature and enhance the external validity of the data collected in the GN registry. Our findings lead us to propose areas that warrant further research, which, in turn, could contribute to the promotion of policies aimed at reducing the rate of cesarean sections. This, in turn, may play a positive role in decreasing complications associated with VBAC and RCB.

### Strengths and limitations

This analysis has some limitations due to its reliance on the secondary analysis of quantitative data, lacking additional context regarding women’s preferences for their mode of birth and their labor experiences in cases of failed trial of labor after cesarean, also to discuss the complications related to VBAC and RCB. However, the study’s strengths lie in its utilization of large sample sizes drawn from diverse global populations, reflecting the increasing rates of C-sections. Moreover, the study’s unique contribution emerges from its broader scope, standing in contrast to narrower, single-site, or regional analyses.

## Data Availability

The datasets used and/or analyzed during the current study are available from the corresponding author on reasonable request.

## Abbreviations

BMI: Body max Index
GN: Global Network for Women’s and Children’s Health Research
MNHR: the Maternal and Newborn Health Registry
RCB: Repeat Cesarean Birth
VBAC: Vaginal Birth After Cesarean
